# Suppression of Tumor Growth and Angiogenesis by a Specific Antagonist of the Cell-Surface Expressed Nucleolin

**DOI:** 10.1371/journal.pone.0002518

**Published:** 2008-06-18

**Authors:** Damien Destouches, Diala El Khoury, Yamina Hamma-Kourbali, Bernard Krust, Patricia Albanese, Panagiotis Katsoris, Gilles Guichard, Jean Paul Briand, José Courty, Ara G. Hovanessian

**Affiliations:** 1 CNRS UMR 7149, Université Paris-Est, Créteil, France; 2 CNRS UPR 2228, Université Paris Descartes, Paris, France; 3 Laboratory of Molecular Pharmacology, University of Patras, Patras, Greece; 4 CNRS UPR 9021, Institut de Biologie Moléculaire et Cellulaire, Strasbourg, France; University of Helsinki, Finland

## Abstract

**Background:**

Emerging evidences suggest that nucleolin expressed on the cell surface is implicated in growth of tumor cells and angiogenesis. Nucleolin is one of the major proteins of the nucleolus, but it is also expressed on the cell surface where is serves as a binding protein for variety of ligands implicated in cell proliferation, differentiation, adhesion, mitogenesis and angiogenesis.

**Methodology/Principal Findings:**

By using a specific antagonist that binds the C-terminal tail of nucleolin, the HB-19 pseudopeptide, here we show that the growth of tumor cells and angiogenesis are suppressed in various *in vitro* and *in vivo* experimental models. HB-19 inhibited colony formation in soft agar of tumor cell lines, impaired migration of endothelial cells and formation of capillary-like structures in collagen gel, and reduced blood vessel branching in the chick embryo chorioallantoic membrane. In athymic nude mice, HB-19 treatment markedly suppressed the progression of established human breast tumor cell xenografts in nude mice, and in some cases eliminated measurable tumors while displaying no toxicity to normal tissue. This potent antitumoral effect is attributed to the direct inhibitory action of HB-19 on both tumor and endothelial cells by blocking and down regulating surface nucleolin, but without any apparent effect on nucleolar nucleolin.

**Conclusion/Significance:**

Our results illustrate the dual inhibitory action of HB-19 on the tumor development and the neovascularization process, thus validating the cell-surface expressed nucleolin as a strategic target for an effective cancer drug. Consequently, the HB-19 pseudopeptide provides a unique candidate to consider for innovative cancer therapy.

## Introduction

Nucleolin is an abundant RNA- and protein-binding protein ubiquitously expressed in exponentially growing eukaryotic cells. It is found at several locations in cells: in the nucleolus, it controls many aspects of DNA and RNA metabolism; in the cytoplasm it shuttles proteins into the nucleus and provides a posttranscriptional regulation of strategic mRNAs; and on the cell surface where it serves as an attachment protein for several ligands from growth factors to virus particles [1], [2]. Nucleolin contains three main structural domains: N-terminal region containing several long stretches of acidic residues; central globular domain containing four RNA binding sites; C-terminal domain containing nine repeats of the tripeptide arginine-glycine-glycine (RGG domain). Surface and cytoplasmic nucleolin are differentiated from nuclear nucleolin by a slight shift in their isoelectric point, which could reflect glycosylation of surface/cytoplasmic nucleolin [3], [4].

Since the first report of surface expression of nucleolin in hepatocarcinoma cells, enhanced expression of nucleolin was observed on the surface of tumor and endothelial cells, and *in vivo* in angiogenic endothelial cells within the tumor vasculature [2], [5], [6]. By electron and confocal microscopy studies, we confirmed surface expression of nucleolin and its indirect association with intracellular actin cytoskeleton [3]. An actin based motor protein, the nonmuscle myosin heavy chain 9, could serve as a physical linker between surface nucleolin and actin [7]. Upon stimulation of cell proliferation, cytoplasmic nucleolin is translocated to the surface through a temperature-dependent but unconventional secretory pathway [3]. Surface nucleolin serves as a low affinity receptor for HIV-1 and various growth factors that interact with the RGG domain of nucleolin, such as midkine, pleiotrophin (PTN) and lactoferrin [8]–[11]. Binding of these ligands results in clustering of cell-surface nucleolin in lipid raft membrane microdomains before endocytosis of the ligand-nucleolin complex by an active process [9], [12]. Accordingly, surface nucleolin could shuttle ligands between the cell surface and the nucleus thus act as a mediator for the extracellular regulation of nuclear events [11], [13], [14].

The importance of cell-surface nucleolin in cancer biology is highlighted by many studies showing that ligands of nucleolin play critical role in tumorigenesis and angiogenesis [15], [16]. For example, among surface nucleolin binding growth factors: midkine and PTN can transform cells, whereas on endothelial cells they exert both mitogenic and angiogenic effect [17]. Laminin-1 that induces differentiation of cells binds surface nucleolin, while urokinase that is implicated in mechanisms regulating pericellular proteolysis, cell-surface adhesion, and mitogenesis binds and is co-internalized with surface nucleolin [14], [18], [19]. Hepatocyte growth factor that regulates angiogenesis, invasion and growth of carcinoma cells uses surface nucleolin as an alternative receptor [20]. The tumor homing peptide F3 that binds both endothelial and tumor cells is internalized via surface nucleolin, while endostatin that inhibits angiogenesis binds nucleolin on the surface of endothelial cells before translocation to the nucleus [6], [21]. Finally, expression of nucleolin is enhanced on the surface of endothelial cells upon stimulation with the vascular endothelial growth factor (VEGF), and functional blockade or down-regulation of surface nucleolin in endothelial cells inhibits migration of endothelial cells and prevents capillary-tubule formation [7].

In addition to its function at the cell surface, nucleolin present in the cytoplasm binds 3′-untranslated region in the mRNA of matrix-metalloproteinase-9 (MMP-9) and *bcl-2* oncogene, a process that is necessary for the stability and translational efficiency of these mRNAs [22]–[24]. Nucleolin-binding to MMP-9 mRNA increases the production of the enzyme that by degrading extracellular matrix components promotes tumor metastasis, whereas in B-cell chronic lymphocytic leukemia cells the increased levels of cytoplasmic nucleolin is directly related to overexpression of the *bcl-2* oncogene that blocks apoptosis. Finally, nucleolin has been reported to reduce the level of tumor suppressor protein p53 in breast cancer cells, cooperate with Ras oncogene in transforming primary rat fibroblast and associate with the tumor suppressor retinoblastoma protein to trigger carcinogenesis in human papillomavirus 18-induced cervical carcinoma [25]–[27].

**Figure 1:**
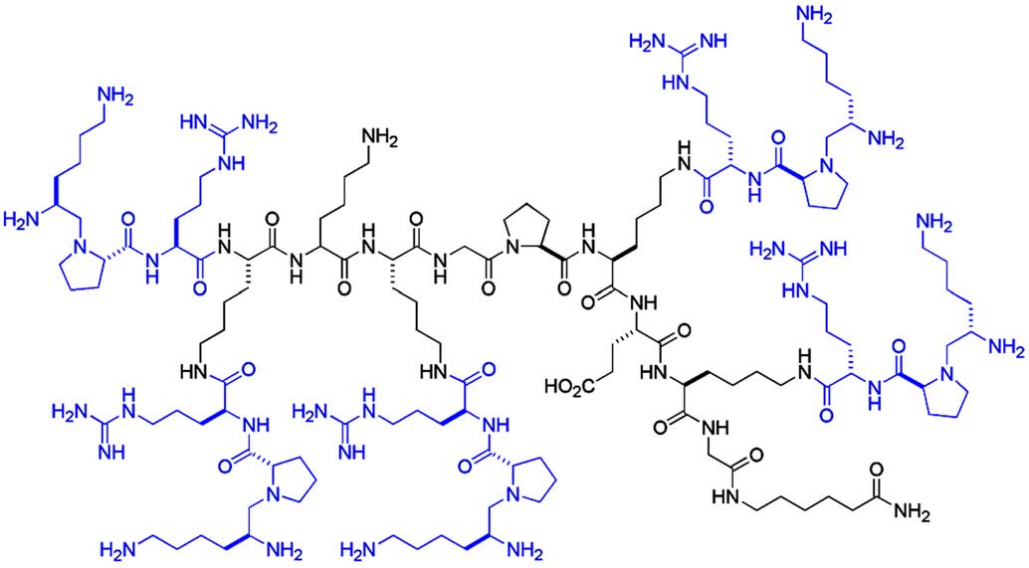
Molecular structure of the multivalent HB-19 pseudopeptide. HB-19 presents pentavalently the pseudo-tripeptide Lysψ(CH_2_N)-Pro-Arg coupled to a template (_H2N_Lys-Lys-Lys-Gly-Pro-Lys-Glu-Lys-Ahx_CONH2_); ψ(CH_2_N) stands for a reduced peptide bond between Lys and Pro residues. The tripeptide is assembled on the α-NH_2_ of the template and the ε-NH_2_ groups of the four lysine residues indicated in bold. No apparent modification is observed for the HPLC profile of HB-19 after five days of incubation at 37°C in normal human serum, thus illustrating its resistance to degradation by serum proteases.

These observations suggested that cell-surface nucleolin is a potential target for the action of anticancer drugs. For this purpose, we used the HB-19 pseudopeptide that binds the C-terminal RGG domain of cell-surface expressed nucleolin and blocks its function as a low affinity receptor for various ligands [8]–[11], [28]. HB-19 presents pentavalently the tripeptide Kψ(CH_2_N)PR in which the reduced peptide bond provides high stability against serum proteases (Figure 1). HB-19 forms a stable complex with the cell-surface expressed nucleolin and is internalized by an active process. This leads to down regulation of surface nucleolin without any apparent effect on nuclear nucleolin, since internalized HB-19 does not cross the nuclear membrane. Here we provide evidence to show that HB-19 inhibits growth of tumor cells and formation of blood vessels in variety of experimental models. Significantly, HB-19 treatment markedly suppressed the progression of established human breast tumor cells xenografted in athymic nude mice, and in some cases eliminated measurable tumors while displaying no toxicity to normal tissue. This potent antitumoral effect appears to be the consequence of the direct dual action of HB-19 on tumor and endothelial cells resulting in the inhibition of multiplication of tumor cells, and the inhibition of proliferation and migration of endothelial cells. Our studies demonstrate the unique properties of HB-19 and provide the rationale for its future clinical evaluation in cancer therapy.

## Results

### The HB-19 pseudopeptide binds the cell-surface expressed nucleolin

Cell-surface expressed nucleolin is solubilized along cytoplasmic nucleolin during preparation of nucleus-free cell extracts using a nonionic detergent-containing solution in the presence of MgCl_2_. Consequently, nucleus-free subcellular fractions referred to as surface/cytoplasmic extracts contain both surface and cytoplasmic nucleolin in contrast to nuclear extracts that contain nucleolin from the nucleoli. Surface/cytoplasmic and nuclear nucleolin have distinct isoelectric point values [3]. The fact that HB-19 forms a stable complex with the cell-surface expressed nucleolin, incubation of cells with biotinylated HB-19 (HB-19/Btn) followed by purification of surface/cytoplasmic extracts using avidin-agarose provides an efficient method for purification surface nucleolin and monitoring its expression [8].

**Figure 2:**
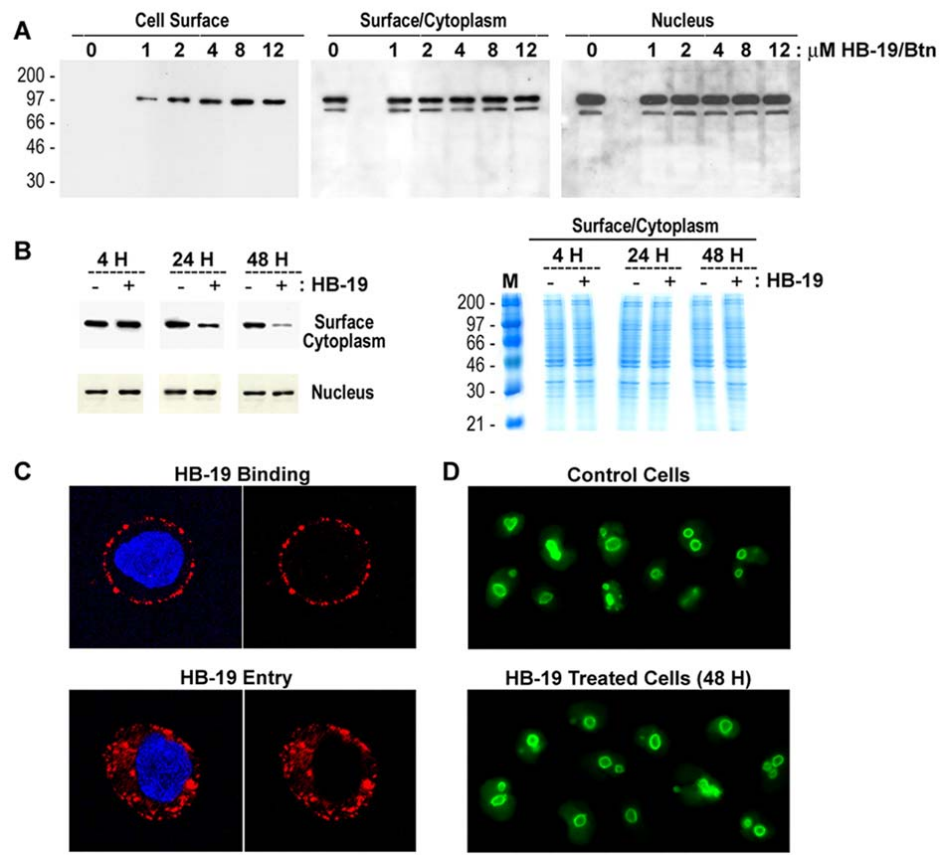
HB-19 binds nucleolin expressed on the surface of tumor cells and causes reduction of cytoplasmic/surface but not nuclear nucleolin. (A) HB-19/Btn forms a stable complex with nucleolin expressed on the surface of cells. MDA-MB-231 cells were incubated (45 min, 20°C) with 0, 1, 2, 4, 8 and 12 µM of HB-19/Btn before preparation of surface/cytoplasmic (i.e., nucleus-free) and nuclear extracts [8], [28]. Samples of surface nucleolin (purified using surface/cytoplasmic extracts from 2×10^7^ cells) and crude surface/cytoplasmic and nuclear extracts (from 2×10^6^ cells) were analyzed by immunoblotting for the detection of nucleolin using mAb D3. (B) Specific reduction of surface/cytoplasmic nucleolin in HB-19 treated cells. MDA-MB-231 cells were cultured (at 37°C) with 10 µM of HB-19 for 4, 24 and 48 hours before preparation of cytoplasmic and nuclear extracts. Material from 2×10^6^ cells (Untreated or HB-19 treated cells as indicated − or + signs, respectively) was analyzed by immunoblotting for the detection of nucleolin. Sections of the gel at the position of the nucleolin bands are presented. The right panel shows the profile of proteins in the Nuclear-free cell extract (Surface/Cytoplasm) of the PAGE-SDS gel stained with Brilliant Blue G-Colloidal Concentrate. Lane M shows the electrophoretic mobility of protein markers. The intensity of nucleolin protein bands quantified by using the NIH image software indicated 75% and 93% reduction of surface/cytoplasmic nucleolin in HB-19 treated cells at 24 and 48 hours, respectively, compared to the corresponding untreated cells. (C) HB-19 binds the cell surface and enters in the cytoplasm but not the nucleus. MDA-MB-231 cells were incubated at 20°C (for HB-19 Binding) or 37°C (for HB-19 Entry) with 5 µM of HB-19/Btn for 1 hour before fixation in PFA or PFA-Triton, respectively. Fixed cells were successively incubated with rabbit anti-biotin and goat Texas Red-conjugated anti-rabbit IgG and the nuclei were colored with DAPI. Scans corresponding to the cross-section of cells are shown, each with or without the DAPI. (D) Nucleolar nucleolin is not affected in HB-19 treated cells. MDA-MB-231 cells cultured in the absence (Control Cells) or presence of 10 µM of HB-19 for 48 hours (HB-19 Treated Cells) were fixed in PFA-Triton and processed for fluorescence microscopy. Nucleolin was revealed by mAb D3 and FITC-conjugated goat anti-mouse IgG.

**Figure 3:**
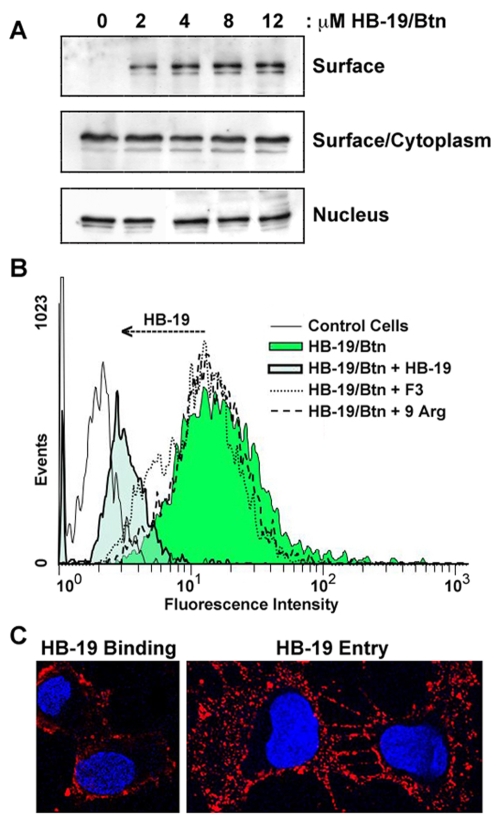
Specific binding and entry of HB-19 in endothelial cells. (A) HB-19/Btn forms a stable complex with nucleolin expressed on the surface of HUVECs. Cells were incubated (45 min, 20°C) with 0, 1, 2, 4, 8 and 12 µM of HB-19/Btn before preparation of surface/cytoplasmic and nuclear extracts and recovery of surface nucleolin from the surface/cytoplasmic extracts as in Figure 2A. Material extracted from 2×10^7^ and 2×10^6^ cells was analyzed in panels Surface and Surface/Cytoplasm or Nucleus, respectively. Immunoblotting was with the anti-nucleolin mAb D3. (B) The specific binding of HB-19/Btn to HUVECs. The binding of 1 µM HB-19/Btn was studied at 4 °C by FACScan analysis. To show the specificity of binding, the reaction was carried out in the absence or presence of 50 µM HB-19, F3, or 9Arg peptides. The ordinate gives the relative cell number, whereas the abscissa gives the relative fluorescence intensity. (C) HB-19 entry in HUVECs. Cells were incubated with 5 µM of HB-19/Btn (1 hour, 37°C) before fixation in PFA (for surface binding) or PFA-Triton (for entry), respectively. Cells were then successively incubated with rabbit anti-biotin antibodies and goat Texas Red-conjugated anti-rabbit IgG, and processed for confocal microscopy. The scans of cells toward the middle cell layer are presented (with the DAPI stained nuclei).

The interaction of HB-19 with surface nucleolin was investigated using human breast cancer MDA-MB-231 tumor cells and human umbilical vein endothelial cells (HUVECs). In both types of cells, we showed that iodinylated HB-19 binds cells in a dose-dependent manner reaching saturation at about 1–2 µM concentration. This binding is specific as it is prevented by unlabeled HB-19 with Kd value of 312 nM and 825 nM in MDA-MB-231 cells and HUVECS, respectively (data not shown). Similarly, HB-19/Btn binds and forms a stable complex with surface nucleolin in a dose-dependent manner in both types of cells, with maximum binding occurring at 8–12 µM concentration (Figures 2A, 3A). By FACScan analysis, we showed that HB-19/Btn binding to HUVECs is specific since it is prevented by the excess HB-19 but not by the basic 9Arg peptide or the F3 peptide (Figure 3B), which is reported to bind the acidic amino acid region located at the N-terminal part of nucleolin [6].

Following binding to surface nucleolin, HB-19 is internalized and is concentrated in the cytoplasm without translocation into the nucleus (Figures 2C, 3C). During this time, HB-19 exerts a differential inhibitory effect on the expression of cytoplasmic/surface nucleolin compared to nuclear nucleolin as shown by the marked reduction of nucleolin in cytoplasmic extracts of cells but without apparent modification of either level (Figure 2B) or nucleolar localization of nuclear nucleolin (Figure 2D), even after 24–48 hours of HB-19 treatment. The fact that expression of other cytoplasmic proteins is not affected in HB-19 treated cells indicates that the marked drop of cytoplasmic/surface nucleolin is a specific and a selective effect (Figure 2B).

HB-19 is internalized at 37°C but at 20°C it remains attached to the cell surface without entering the cytoplasm, thus indicating that it uses an active internalization process.

### *In vitro* antitumorigenic activity of HB-19

**Figure 4:**
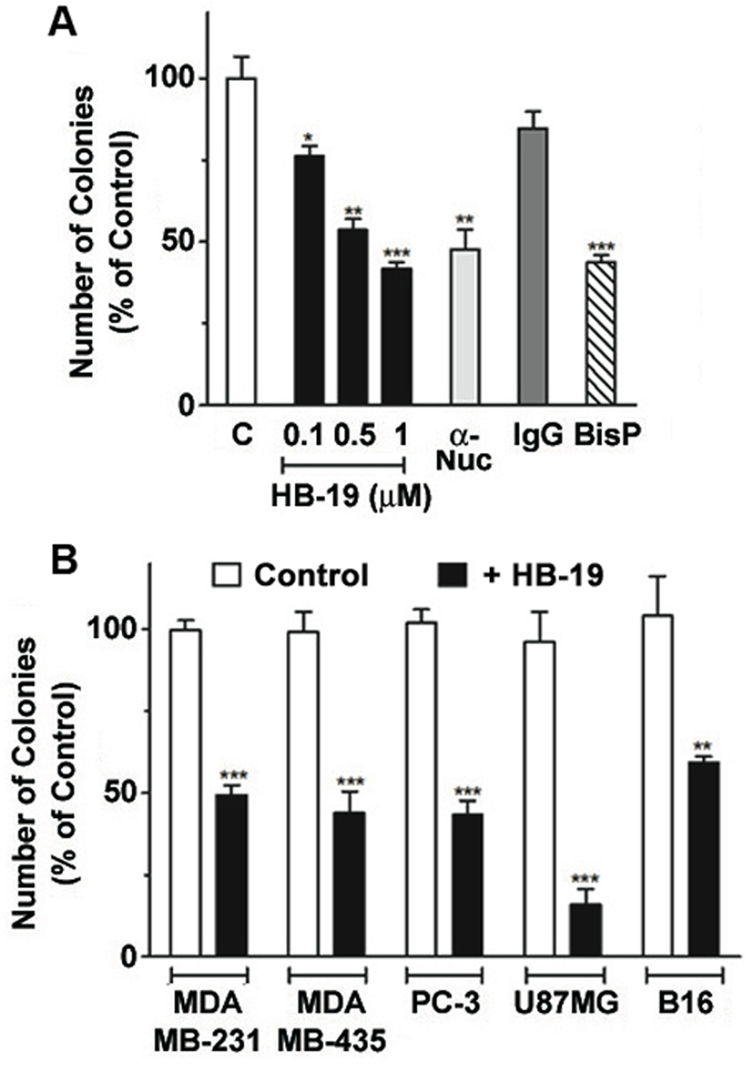
HB-19 inhibits colony formation in soft agar by tumor cell lines. (A) MDA-MB-231 cells in culture medium were seeded in triplicate in the absence (histogram C) or presence of 0.1, 0.5 and 1 of µM HB-19, or 0.1 µM of the anti-nucleolin mAb MS-3 or control IgG, or 10 µM bisphosphonate (BisP). (B) Various tumor cell lines (as indicated) in culture medium were seeded in triplicate in the absence (histogram Control) or presence of 5 µM HB-19. After 10–21 days, colonies with diameters greater than 50 µm were scored as positive. Statistical significance: *0.01<p<0.1, **p<0.01, ***p<0.001.

The inhibitory action of HB-19 on tumor cell replication was evaluated in soft-agar colony-forming assay where inhibition of colony formation is considered a stringent test of the anticancer activity of a compound. Both HB-19 and anti-nucleolin monoclonal antibody (mAb) reduced significantly colony-forming capacity of MDA-MB-231 cells with 50% reduction occurring at 1 µM of HB-19 (Figure 4A). Under similar experimental conditions, 60% reduction occurred at 10 µM bisphosphonate that induces apoptosis. Consistently, at 5 µM of HB-19, colony formation was reduced markedly in human carcinoma cells of different origins, such as breast cancer (MDA-MB-231 and MDA-MB-435), prostatic adenocarcinoma (PC3), glioblastoma (U87MG), and murine melanoma cells (B16), thus illustrating the antitumoral potential of HB-19 (Figure 4B).

**Figure 5:**
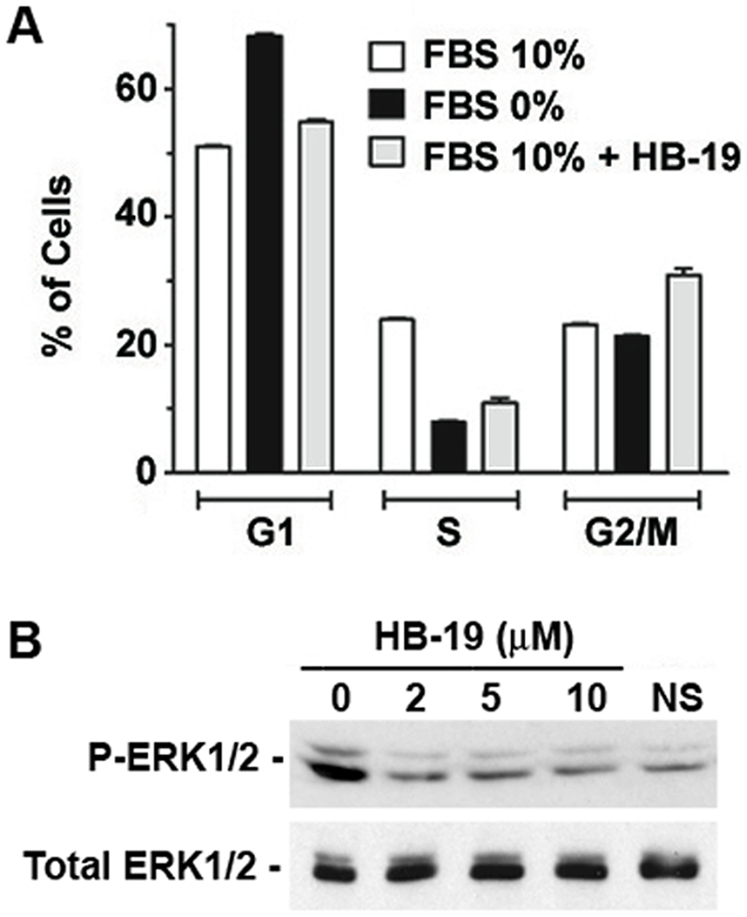
Cell cycle perturbations induced by HB-19 treatment. (A) Analysis of cell cycle parameters in HB-19 treated cells. MDA-MB-231 cells were cultured for 48 hours in medium without FBS (starvation) or in medium containing 10% FBS supplemented or not with 10 µM HB-19. DNA synthesis was quantified after BrdU incorporation and staining with anti-BrdU antibody and 7-AAD, by FACScan analysis. The histograms indicate the relative amount of cells in G1, S and G2/M cell phases. (B) HB-19 treatment inhibits serum-induced phosphorylation of ERK1/2. Serum starved MDA-MB-231 cells were stimulated with 10% FBS in the absence or presence of 2, 5, and 10 µM of HB-19. Five minutes after serum stimulation, cells were lysed directly in electrophoresis sample buffer and processed for immunoblotting using anti-phospho-p42/44 ERK1/2 and anti-p42/44 ERK antibodies. NS stands for non-stimulated cells.

In spite of marked inhibitory effect on tumor cell proliferation, apoptosis was not observed in HB-19 treated cells (not shown). We therefore investigated cell cycle perturbations induced by HB-19 treatment in MDA-MB-231 cells and compared the results with perturbations induced by serum starvation (Figure 5A). Compared to untreated cells, HB-19 treatment resulted a 62% decrease of cells in the S phase with an increase of 31% and 9% in the G2/M and G1 phases, respectively. In serum starved cells, a marked reduction (70%) of cells occurred as expected in the S phase, which was accompanied by 25% increase of cells in the G1 phase of the cell cycle but the proportion of cells in the G2/M phase was not modified. As the S phase represents the period in which cells replicate their DNA, the marked reduction in the proportion of HB-19 treated cells in the S phase is consistent with its inhibitory effect on tumor cell multiplication in soft agar. Recently, depletion of total cellular nucleolin (surface/cytoplasmic+nuclear nucleolin) by siRNA was shown to result in cell growth arrest and accumulation of cells in the G2 phase without affecting the proportion of cells in the S phase [29], which is in contrast to HB-19 that causes marked reduction in the S phase. These observations suggest that surface and nuclear nucleolin can also be differentiated by their mechanism of action on the cell cycle. By blocking surface nucleolin, HB-19 could perturb signaling events during induction of cell proliferation in response to various stimuli, thus illustrating that surface nucleolin could be implicated in mechanisms that link extracellular signals with intracellular signaling [30]. To illustrate this, we tested the effect of HB-19 on the activation of the extracellular signal-regulated ERK1/2, one of the well-characterized mitogen-activated protein kinases. Figure 5B shows that blockade of surface nucleolin by HB-19 in MDA-MB-231 cells prevents enhanced phosphorylation of ERK1/ERK2 occurring at 5 min in response to serum stimulation of cells.

### Inhibition of angiogenesis by targeting surface nucleolin

**Figure 6:**
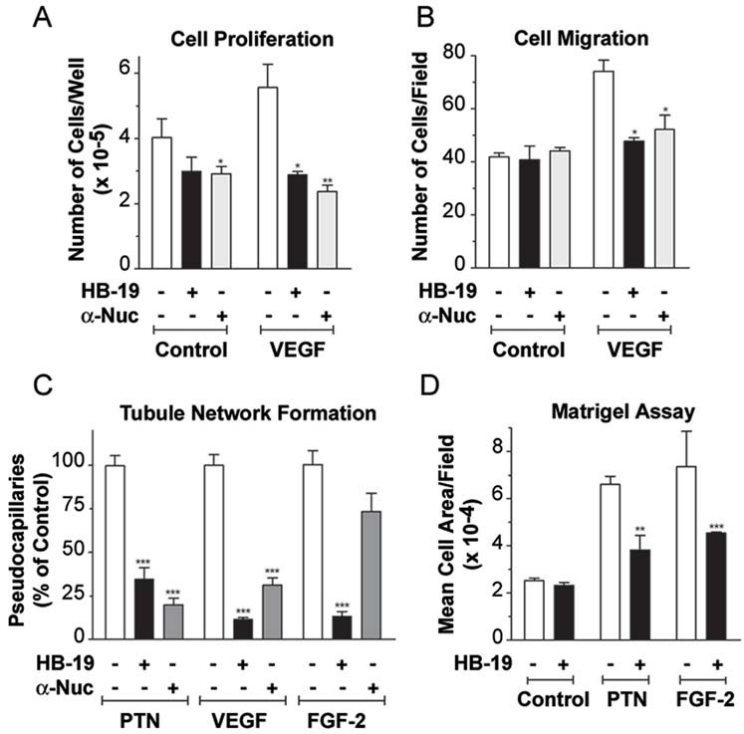
Inhibition of *in vitro* and *in vivo* angiogenesis by HB-19. (A) HB-19 inhibits proliferation of HUVECs. Twenty-four hours after seeding HUVECs in 2% FBS, cells were stimulated by 0.25 nM VEGF_165_, in the absence or presence of 1 µM HB-19 or 0.1 µM anti-nucleolin mAb MS-3 as indicated. After 72 h, cell number was determined by crystal violet staining [47]. The data are reported as the mean of triplicate samples. (B) HB-19 inhibits migration of HUVECs. Cell migration was studied using a modified Boyden chamber. Cells were incubated (4 h at 37°C) with VEGF_165_, HB-19, or anti-nucleolin mAb MS-3 as above. Cells that migrated through the pore to the lower filter surface were counted and are shown as number per microscopic field at ×100 magnification. The data are the mean of three independent high-power fields/well performed in duplicate in two independent experiments. (C) HB-19 inhibits tubular network structure formation in collagen. Aortic endothelial (ABAE) cells in the absence or presence of 3 nM PTN, 0.5 nM VEGF_165_, and 3 nM FGF-2 were seeded on a three-dimensional collagen gel in complete medium. Treatment with HB-19 or anti-nucleolin mAb MS-3 was for 3 days after cell plating. Tubular network structures were quantified using phase contrast microscopy (×100). The ordinate gives the number of pseudocapillaries corresponding to the means of three randomly chosen fields/well from three wells. (D) HB-19 inhibits ex vivo angiogenic activity of growth factors in a Matrigel plug model. Liquid Matrigel was subcutaneously injected into the flank of Swiss mice in the absence or presence of 5 nM PTN, 10 nM FGF-2, and 1 µM HB-19. Quantification of endothelial cell invasion into the Matrigel was determined and is expressed as a mean of five fields per section from 3 Matrigel-plug sections per mouse. The results are expressed as the mean of five mice per group. Statistical significance: *p<0.05, **p<0.01, ***p<0.001.

In an *in vitro* proliferation and migration assay of VEGF stimulated HUVECs, HB-19 and anti-nucleolin mAb inhibited dramatically both of these events down to levels comparable to that of unstimulated cells (Figure 6A, 6B). In an *in vitro* model of endothelial cell differentiation on a three-dimensional collagen gel, HB-19 and anti-nucleolin mAb inhibited formation of capillary-like branched structures induced by angiogenic growth factors PTN and VEGF (Figure 6C). On the other hand, the inhibitory effect of anti-nucleolin antibody was much less pronounced compared to HB-19 on such tubular network formation when FGF-2 was used as an inducer (Figure 6C). This difference could be due to the inhibitory mechanism of action of HB-19 on surface nucleolin compared to that of the anti-nucleolin mAb.

**Figure 7:**
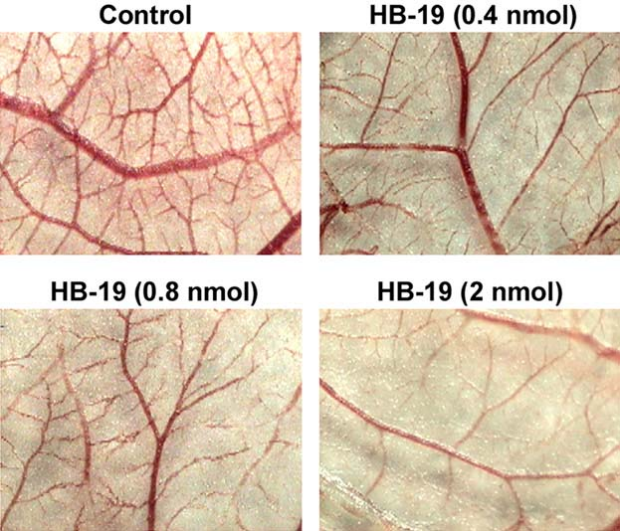
Inhibition of *in vivo* angiogenesis by HB-19 in chick embryo chorioallantoic membrane (CAM) assay. Macroscopic observation of the angiogenic response induced by gelatin sponges (1 cm^2^), soaked with 40 µl of PBS (Control) or 40 µl PBS containing HB-19 at concentrations of 10, 20 and 50 µM (corresponding to 0.4, 0.8 and 2 nmols of HB-19, respectively). After 48 h, CAMs were fixed and excised from the eggs . Photographs were taken and the total length of the vessels was measured using the Image PC image-analysis software (Scion Corporation, USA)[48]. Each sample was tested three times using 15–20 eggs for each data point. The relative percent inhibition (P<0.001) of angiogenesis compared to the control was 27, 36 and 51% in the presence of 0.4, 0.8 and 2 nmols of HB-19, respectively.

The antiangiogenic potential of HB-19 was further evaluated in the mouse Matrigel plug assay using growth factors PTN and FGF-2. One week after subcutaneous injection of Matrigel into the flank of mice, angiogenesis was assessed by scoring the vascular density into the Matrigel plug. HB-19 treatment significantly inhibited the *ex vivo* angiogenesis induced by FGF-2 or PTN as demonstrated by reduced endothelial-cell infiltration into the Matrigel plug (Figure 6D). Finally, the antiangiogenic potency of HB-19 was confirmed *in vivo* using chicken embryo chorioallantoic membrane (CAM) angiogenesis model [31], in which HB-19 impaired significantly blood vessel branching and development in a dose-dependent manner (Figure 7). Our results illustrate the potent antiangiogenic activity of HB-19, and consistent with previous reports they confirm the function of surface nucleolin in the development of the vascular network [7], [15], [16].

### *In vivo* antitumorigenic activity of HB-19

**Figure 8:**
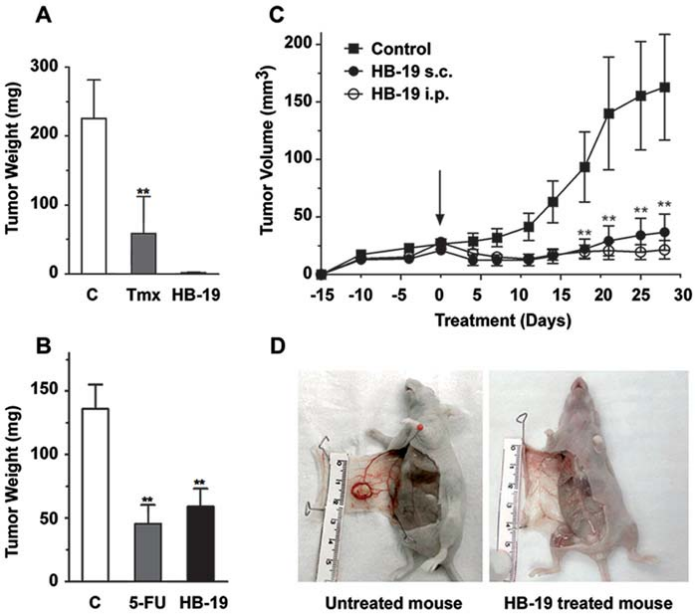
HB-19 inhibits tumor growth in the nude mice. (A) HB-19 inhibits the growth of MDA-MB-231 tumor-cell xenografts. Cells (2×10^6^) were injected subcutaneously into the right flank of female nude mice. Two weeks later, mice with a palpable tumor of approximately 40 mm^3^ in volume were randomly separated into three groups (n = 5) and were given peritumoral injections 3 times/week of 0.1 ml PBS alone (Control), HB-19 (5 mg/kg), or Tamoxifen (Tmx) 10 mg/kg) for 6 weeks. (B) HB-19 inhibits the growth of MDA-MB-435 tumor-cell xenografts. Cells (1×10^6^) were injected in the mammary fat pad of female nude mice. Two weeks later, mice with a palpable tumor were randomly separated into three groups (n = 10) and were given intraperitoneal injections 3 times/week of 0.1 ml PBS alone (Control), HB-19 (5 mg/kg), or 5-fluouracil (5-FU, 40 mg/kg) for 8 weeks. At the end of each experiment (in A and B), mice were sacrificed and the tumors were excised and weighed. The results are presented as the mean weight ±standard deviation (±S.D.) obtained from the number of mice in each group. (C, D) Inhibition of tumor development in mice treated by intraperitoneal (i.p.) and subcutaneous (s.c.) administration of HB-19. MDA-MB-231 tumor bearing mice in three groups (n = 10) were treated with HB-19 (10 mg/kg) by i.p. or s.c. injections, 3 times/week for 28 days. The arrow at day 0 shows initiation of HB-19 treatment. Panel D shows MDA-MB-231 tumor bearing mice, untreated control and HB-19 treated (i.p. injection). Statistical significance: *p<0.05, **p<0.01, ***p<0.001.

Inhibitory action of HB-19 was tested against established human breast carcinoma xenografts in the athymic nude mice. For this purpose, mice were inoculated subcutaneously with MDA-MB-231 or MDA-MB-435 cells that proliferate into palpable tumors in 2 weeks. Tumor bearing mice were then treated with HB-19 (Figure 8A, B) or control antitumoral reagents such as tamoxifen and 5-fluouracil (5-FU). The mice were administered 3 times per week with various drugs, during which time tumor-volume was measured by a caliper every third or fourth day. At the end of experiment, tumor-bearing mice were sacrificed, blood was collected for analysis, and the liver, lungs and spleen were removed for histological analysis. Besides a marked suppression of tumor development in HB-19 treated mice, autopsy revealed no apparent effect on various tissues compared to control mice injected with PBS alone. Similarly, HB-19 treatment had no effect on body weight and on blood cell counts, nor induced any evidence of toxicity such as diarrhea, infection, weakness, or lethargy (data not shown).

**Figure 9:**
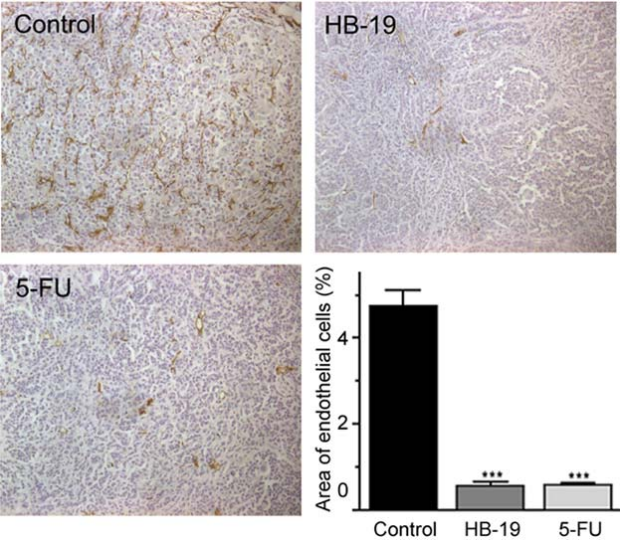
Reduced density of blood vessels in HB-19 treated tumor-bearing mice. Sections of tumors recovered from mice, untreated control and treated with either HB-19 or 5-FU (experiment described in Figure 8B) were stained with antibodies against the CD31 endothelial marker and analyzed by fluorescence microscopy. Representative macroscopic image (magnification 200×) from each group of mice shows the marked reduction of blood vessels in the tumors recovered from HB-19 or 5-FU treated animals. Angiogenesis was quantified by image analysis of CD31-labeled endothelial cells. The graph shows the mean areas ±standard deviation obtained from control and treated mice. ***p<0.001.

In the ectopic MDA-MB-231 xenograft model, peritumoral administration of HB-19 strongly inhibited tumor growth and in some cases eliminated measurable tumors (Figure 8A). In addition, the results show that the antitumoral activity of HB-19 is superior to the conventional cancer drug tamoxifen. Indeed, in tumor bearing mice, treatment with HB-19 (5 mg/kg) inhibited >95% tumor growth, whereas treatment with tamoxifen (10 mg/kg) reduced tumor mass by only 80%. In the orthotopic MDA-MB-435 xenograft model, intraperitoneal administration of HB-19 impaired tumor growth by 57% compared to 66% by the widely used chemotherapeutic drug 5-FU (Figure 8B). Similarly, HB-19 and 5-FU treatment significantly inhibited tumor vascularization compared to the untreated tumors as revealed by immunohistochemical analysis using antibodies against the CD31 endothelial marker (Figure 9). On the other hand, 5-FU but not HB-19 was toxic causing a significant degree of leucopenia with a 55% reduction in the number of lymphocytes. HB-19 treatment had no effect on the number of platelets, erythrocytes, and leukocytes (Table 1).

**Table 1:** HB-19 treatment has no effect on blood cell number.

Cells	Untreated mice	5-FU treated mice	HB-19 treated mice
**Platelets^1^**	1041±91	1042±59	1037± 225
**Erythrocytes^2^**	8.73±0.26	7.60±0.30***	8.31±1.05
**Leukocytes^1^**	3.05±1.29	1.73±0.37 *	3.36±1.28
**Lymphocytes^1^**	1.84±0.78	0.83±0.22 **	1.98±1.01
**Monocytes^1^**	0.20±0.09	0.22±0.09	0.23±0.07
**Neutrophils^1^**	0.89±0.39	0.53±0.15 *	1.03±0.44
**Eosinophils^1^**	0.01±0.02	0.05±0.04	0.03±0.04
**Basophils^1^**	0.10±0.06	0.10±0.04	0.09±0.04

MDA-MB-231 tumor bearing mice untreated, and treated with 5-FU or HB-19 were as described in Figure 8B for 8 weeks. Blood samples collected in EDTA were processed using an automated flow cytometric blood cell counter (^1^ ×10^3^/µl; ^2^ ×10^6^/µl). The results are presented as the mean areas ±standard deviation for each mice group (n = 10). Data from untreated and treated group were compared and analyzed using the paired t-test to obtain *p* value: ^*^ p<0.05, ^**^ p<0.01, ^***^ p<0.0001.

Finally, we investigated the contribution of intraperitoneal versus subcutaneous route of injection on the activity of HB-19. Figure 8C shows the progression of MDA-MB-231 tumor xenografts in the nude mice, untreated or treated with HB-19 by injections every 3 or 4 days for a period of 30 days. The antitumoral activity of HB-19 administered by either route was highly effective with more than 95% inhibition in tumor volume. Complete eradication of the tumor was observed in several mice treated intraperitoneally with HB-19 (Figure 8C and 8D).

## Discussion

Several reports have suggested that cell-surface nucleolin is implicated in growth of tumor cells and angiogenesis [2], [15], [16]. The results presented herein provide evidence for the first time that the specific antagonist of surface nucleolin, the HB-19 pseudopeptide, suppresses both of these events. HB-19 inhibits colony formation in soft agar of various tumor cells lines and impairs several endothelial cell functions involved in angiogenesis, such as cell proliferation and migration, tubule network formation, and neovascularization. In the tumor bearing nude mice, HB-19 suppresses markedly progression of established human tumor xenografts, and in some cases results complete eradication of the tumor without any apparent toxicity. Tumor growth and progression is dependent on neovascularization, which is orchestrated by down regulation of antiangiogenic factors and upregulated proangiogenic factors associated with carcinogenesis [32]. Moreover, several growth factors have the capacity to stimulate concurrently tumor cell growth and endothelial cell function [33]. In spite of such complexity, targeting surface nucleolin with HB-19 is effective for inhibition of tumor cell proliferation and impairment of angiogenesis. Therefore functional blockade of surface nucleolin seems to result in a general inhibitory mechanism that is not specific to a single pathway or a growth factor implicated in carcinogenesis. Our results illustrate the dual inhibitory action of HB-19 on tumor and endothelial cells, and validate surface nucleolin as an important anticancer target.

The cross-linking of cell surface bound HB-19/Btn with anti-biotin antibodies results in ligand-dependent clustering of surface nucleolin and its colocalization with the pseudopeptide, thus confirming that surface nucleolin is the target of HB-19 [12]. The presence nucleolin on the cell surface is the consequence of active translocation of cytoplasmic nucleolin to the surface upon stimulation of cell proliferation. This latter and fluctuations in the level of surface nucleolin occur in the absence of any apparent effect on nuclear nucleolin. Surface and cytoplasmic nucleolin are characterized by similar isoelectric points with pI values at about 4.5, whereas nuclear nucleolin is composed of several subspecies with pI values between pH 4 and 6 [3], [12], [28]. These observations suggest that expression of surface nucleolin should be differentially regulated compared to its nuclear counterpart. Consistent with this, our results demonstrate that HB-19 causes a selective reduction of surface nucleolin without affecting the level and nucleolar localization of nuclear nucleolin. After binding surface nucleolin, HB-19 enters cells by an active process and accumulates in the cytoplasm but it does not cross the nuclear membrane in contrast to physiological ligands of surface-nucleolin [11], [13], [14]. Consequently, the effect of HB-19 is exerted differentially and specifically via the cell surface expressed nucleolin, which eventually becomes degraded causing reduction of cytoplasmic pools of nucleolin [28].

The mechanism by which down regulation of surface nucleolin by HB-19 results in inhibitory effects on tumor cells and angiogenesis remains to be elucidated. HB-19 binds the RGG domain at the C-terminal end of nucleolin, which is also the site for binding of RNA [34], [35], rDNA [36], subset of ribosomal proteins [37], the urokinase-type plasminogen activator [14], and several growth factors [8]–[11]. The irreversible binding of HB-19 to this RGG domain could then prevent the proper functioning of surface nucleolin thereby exerting its antagonistic action. In view of the implication in tumor growth and angiogenesis, its capacity to bind pathogens and diverse range of ligands including low density lipoproteins [1], [2], [5], it is plausible to suggest that surface nucleolin could function as a scavenger receptor [38]. Recent studies indicate that at the cell surface, nucleolin exists in a 500-kDa complex containing several proteins whose identity is under investigation (A.G.H. and B.K., unpublished results). Consequently, the binding of HB-19 to surface nucleolin could also affect the organization of nucleolin-associated proteins in this 500-kDa complex and thus generate more inhibitory mechanisms.

HB-19 treatment is not toxic *in vitro* and *in vivo*. The lack of translocation of HB-19 to the nucleus and nucleolus probably accounts for its lack of toxicity in cultured cells and in mice, since nuclear nucleolin is involved in many aspects of gene expression [1], [2]. By using β-radio imager whole-body mapping in rats, we have shown that systematically administered HB-19 is rapidly cleared from blood to become distributed selectively in tissues expressing enhanced levels of surface nucleolin, where a significant proportion still persists even after 24 hours in its active form [39]. In spite of its pseudopeptide nature, HB-19 is eliminated gradually by renal glomerular filtration and most of the excreted radioactivity in the urine is in the form of HB-19 metabolites. This could contribute to the lack of toxicity in animals that were treated for several weeks with HB-19. Furthermore, there is a threshold for the tissue uptake of HB-19, thus ruling out any eventual toxic effects at increased doses [39]. A few minutes following intravenous injection of HB-19, a significant proportion of the peptide is recovered in the bone marrow, which harbors endothelial progenitor cells [39], [40]. Antiangiogenic activity of HB-19 therefore, could be the sum of inhibitory action on endothelial precursor cells in the bone marrow as well as endothelial cells in the circulation and in the tumor microenvironment.

Previously, guanosine-rich phosphodiester oligonucleotides (referred to as GROs) and endostatin have been reported to exert antitumoral and antiangiogenic activity, respectively, through interaction with surface nucleolin [21], [41], [42]. Although GROs bind solubilized nucleolin in cell extracts, surface nucleolin does not seem to mediate their internalization into intact cells (A.G.H. unpublished results). Indeed, GROs are internalized rapidly by a passive process and accumulate in the nucleoli in contrast to the active entry of HB-19 and its accumulation in the cytoplasm. At 20°C, GROs still end up in the nucleolus whereas HB-19 remains aggregated at the plasma membrane. In contrast to GROs, endostatin colocalizes with surface nucleolin and is translocated to the nucleus where it inhibits phosphorylation of nuclear nucleolin [21]. The difference between subcellular localization of HB-19 and endostatin could be accounted by the nature of interaction with surface nucleolin, since endostatin but not HB-19 binding to surface nucleolin requires heparan sulfate proteoglycans [21], [28]. In view of these differences and the fact that HB-19 does not cross the nuclear membrane, it appears that GROs and endostatin function preferentially through nuclear nucleolin while HB-19 acts directly on surface nucleolin. This could account for the differences observed in the inhibitory mechanism of action of GROs and endostatin compared to that of HB-19. For example, GROs result in the accumulation of tumor cells in S phase and induce apoptosis [42], whereas HB-19 causes a reduction of cells in S phase without apoptosis. Similarly, endostatin does not directly affect tumor cell growth because of a defect in its internalization [21]. Therefore, the target of anti-nucleolin reagents needs to be characterized in respect to the specificity of their action on surface or nuclear nucleolin.

The dual and direct action of HB-19 on tumor and endothelial cells fulfills the criteria for an efficient anticancer drug, since combination therapy targeting both of these two distinct cell types is considered an efficient strategy in cancer management [43]. HB-19 could also provide a nontoxic drug for the prevention of cancer recurrence and/or metastasis. Moreover, HB-19 could be used as an alternative therapy in cancer patients that develop resistance to chemotherapy [44]. Another advantage of HB-19 over traditional anti-cancer drugs is its capacity to bind surface nucleolin in an irreversible manner under physiological conditions [8] [39], making the half-life of tissue associated HB-19 much longer compared to that of any other cancer drug. Finally, its reproducible synthesis, stability in serum and *in vivo* lack of toxicity make HB-19 a unique drug against tumor growth and angiogenesis, thus providing novel therapeutic opportunities in cancer therapy.

## Materials and Methods

### Peptide constructs and anti-peptide antibodies

HB-19, biotinylated HB-19 (HB-19/Btn: _H2N_Lys-Lys-Lys-Gly-Pro-Lys-Glu-Lys-βAla-Lys(Btn)-βAla_CONH2_; for recovery surface nucleolin) and Tyrosine-coupled HB-19 (HB-19/Tyr: _H2N_Lys-Lys-Lys-Gly-Pro-Lys-Glu-Lys-βAla-Tyr-βAla_CONH2_; for iodination) were synthesized as described previously using solid phase peptide methodology [8], [28]. HB-19/Btn and HB-19/Tyr, [^125^I]HB-19 peptides manifested similar inhibitory activity as HB-19. All peptides were obtained at a high purity (>95%). The control peptides, 9R (composed of nine D-Arg residues) and F3 were as reported [6], [9].

### Reagents

Culture medium and fetal bovine serum (FBS) were supplied by Invitrogen (Cergy Pontoise, France). EBM-2 Bullekit medium was from Biowhittaker (Emerain-ville, France). Matrigel™ was from BD PharMingen (Le Pont de Calais, France). C23 (MS-3) anti-nucleolin mAb was from Santa Cruz biotechnology (France). Mouse control IgG and phosphatase alkaline-conjugated goat anti-rat immunoglobulin G were purchased from Jackson ImmunoResearch (France). Rat anti-mouse CD31 monoclonal antibody (PECAM) was from BD Pharmingen Biosciences (France). Anti-phospho-p42/44 ERK1/2 (Thr202/Tyr204) and anti-p42/44 ERK MAP kinase antibodies were purchased from Santa Cruz Biotechnology, U.S.A. Bisphosphonate, tamoxifen and 5-FU were from Sigma Diagnostics (France). BrdU kit was from (BD Pharmingen Bioscience, France).

### Cell lines and cell culture

The PC3 human prostatic carcinoma cell line, MDA-MB-231 and MDA-MB-435 human breast carcinoma cell lines, U87MG human glioblastoma cell line, and B16 mouse melanoma cells were purchased from ATCC (American Type Culture Collection, Rockville, MD). PC-3 cells were grown in RPMI 1640 supplemented with 10% FBS. MDA-MB-231, MDA-MB-435, U87MG, and B16 cell lines were grown in DMEM 4.5 g/l glucose supplemented with 10% FBS. Human umbilical vein endothelial cells (HUVECs) were provided by Clonetics (Biowhittaker, Emerain-ville, France) and cultured between passages 2 and 5 in EBM-2 Bullekit supplemented medium with 2% FBS. Aortic bovine endothelial cells (ABAE) were cultured in DMEM 1 g/l glucose supplemented with 10% decomplemented FBS. All cultures were grown at 37°C in a humidified atmosphere of 5% CO_2_.

### Preparation of cytoplasmic and nuclear extracts

Cells washed in phosphate-buffered saline (PBS) were lysed in buffer E (20 mM Tris–HCl, pH 7.6, 150 mM NaCl, 5 mM MgCl_2_, 0.2 mM phenylmethylsulfonyl fluoride (PMSF), 5 mM β-mercaptoethanol, aprotinin (1000 U/ml) and 0.5% Triton X-100) and the nuclei were pelleted by centrifugation (1000*g* for 5 min). For the preparation of nuclear extracts, the nuclear pellet was disrupted in buffer I (20 mM Tris–HCl, pH 7.6, 50 mM KCl, 400 mM NaCl, 1 mM EDTA, 0.2 mM PMSF, 5 mM β-mercaptoethanol, aprotinin (1000 U/ml), 1% Triton X-100, and 20% glycerol). The nucleus-free supernatants and the nuclear extracts were then centrifuged at 12,000*g* for 10 min, and the supernatants were stored at −20°C. Aliquots of crude cell extracts were diluted in 2 fold concentrated electrophoresis sample buffer containing SDS, heated and analyzed by SDS- polyacrylamide gel electrophoresis (PAGE). Gels were stained with Brilliant Blue G-Colloidal Concentrate from Sigma.

### Purification of the cell-surface-expressed nucleolin

Cells were passaged in DMEM culture medium containing 10% FCS in 75 cm^2^ flasks. After 2 days, subconfluent cells (about 3×10^6^ cells/flask) were incubated (45 min, 20°C) with 5 µM of HB-19/Btn. After washing extensively in PBS containing 1 mM EDTA (PBS-EDTA), nucleus-free cell extracts were prepared in lysis buffer E. The complex formed between cell-surface expressed nucleolin and HB-19/Btn was isolated by purification of the extracts using NeutrAvidin agarose (100 µl; Pierce Biotechnology) in PBS-EDTA. After 3 hours at 6°C, the avidin-agarose samples were washed extensively with PBS-EDTA. The purified surface nucleolin was eluted in the electrophoresis sample buffer containing SDS and analyzed by SDS–polyacrylamide gel electrophoresis (PAGE). The presence of nucleolin was then revealed by immunoblotting using mAb D3 against nucleolin as described before [8], [28].

### Immunofluorescence and confocal microscopy

Cells were plated 24 hours before the experiment in eight-well glass slides (Lab-Tek Brand; Nalge Nunc International, Naperville, IL). Cells were fixed with either paraformaldehyde (PFA; 3.7%) for membrane staining or PFA/Triton X-100 solution (PFA/Triton) for staining intracellular HB-19/Btn or nucleolar nucleolin [3], [28]. The secondary antibodies were the following: FITC-conjugated goat anti-mouse IgG (Sigma), Rabbit anti-biotin concentrate (IgG fraction; Enzo Dioagnostics, Inc., New York), Texas Red dye-conjugated goat anti-rabbit IgG (Jackson ImmunoResearch Laboratories). In some experiments, the nuclei were colored with 4′,6-diamidino-2-phenylindole (DAPI).

### Colony formation in soft agar

Carcinoma cells (2×10^4^) were mixed in 0.35% top agar, diluted in complete medium (DMEM containing 10% FBS) in the absence or presence of HB-19, and plated onto 0.8% bottom agar into 12-well plates. Cells were treated twice a week during 10 to 21 days. Colonies with diameters greater than 50 µm were scored as positive using a phase contrast microscope equipped with a measuring grid at magnification 100× [45]. The number of colonies was determined by analyzing 5 fields/well from 3 wells and the test was repeated at least twice.

### BrdU incorporation and cell cycle analysis

Cell cycle analysis was performed using the FITC-BrdU flow kit (#559619) kit from BD Bioscience Pharmingen, according to the manufacturer's instructions. Briefly, MDA-MB-231 cells (1×10^5^) were seeded in 12-multiwell and incubated 24 hours for adhesion. Cells were treated with HB-19 for 48 hours and labeled with BrdU (10 µM, 1 hour, 37°C), then they were trypsinized, fixed and treated with DNase (300 µg/ml, 1 hour, 37°C) before staining with anti-BrdU-FITC antibody and 7-AAD reagent. Cells were analyzed on a Becton Dickinson FACScan (fluorescence activated cell sorter), on FL1-H and FL3-H parameters for FITC and 7-AAD fluorescent dye respectively.

### HUVEC endothelial cell chemotactic migration assay

HUVEC migration assays were performed using a 24-well chemotaxis chamber (Transwell, Corning Costar, France). Polycarbonate filters with 8 µm pore size were coated with 10 µg/ml type I collagen R (Serva, Heidelberg, Germany) for 1 hour and dried under sterile air. The EBM-2 medium supplemented with 1% FBS was then placed in the lower chamber and served as chemo-attractant. Cells (10^5^/well) suspended in EBM-2/1% FBS were seeded in the upper compartment in the absence or presence of HB-19 or mAb MS-3. Transwells were incubated for 4 hours at 37°C, after which cells on the upper surface of the filter were removed by wiping with a cotton tip. The filters were then fixed with methanol and stained with May Grünwald Giemsa solution. The cells that had migrated through the pores to the lower filter surface were counted in three random high power fields in each well (magnification 100×). Results are presented as the mean of two independent experiments.

### Angiogenesis tube formation assay

Three-dimensional collagen gels were prepared following the procedure of Montesano with minor modifications [46]. Briefly, 10^5^ aortic endothelial cells (ABAE) per well in 24-well culture plate were seeded on a three-dimensional collagen gel in complete medium. After 24 hours, HB-19 was added daily for three consecutive days. Tubular network structures were then quantified using phase contrast microscopy at 100× magnification [45]. Data are the means of 3 randomly chosen fields/well from 3 wells and the experiment was repeated twice.

### In vivo mouse angiogenesis assay using Matrigel™ plug model

Liquid Matrigel™ (0.3 ml) at 4°C was subcutaneously injected into Swiss mice (n = 5 mice/sample; Janvier, Le Genest St Isle, France) alone or supplemented with PTN (5 nM) or FGF-2 (10 nM), and in the absence or presence of HB-19 (1 µM). The Matrigel™ rapidly formed a single solid gel plug. Mice were sacrificed after 8 days, the skins were pulled back and the intact the plugs were excised and frozen in liquid nitrogen. Sections of 8 µm thickness were cut using a cryostat (Leica), fixed with acetone, and stained with Gomori-Trichrome. Histological slides were analyzed by microscopic observation at 200× magnification to determine the Matrigel™ plug area infiltrated by endothelial cells. Quantification of endothelial cell invasion into the Matrigel™ was determined as a mean of 5 fields per section from 3 plug sections per mouse using NIH Image software [47].

### Chicken embryo chorioallantoic membrane (CAM) assay

Leghorn fertilized eggs (Pindos, Greece) were incubated for 4 days at 37°C. A window was then opened on the eggshell, exposing the CAM. The window was covered with sterile tape, and the eggs were returned to the incubator. On day 9 of embryo development, 20 µl of distilled water alone as a control or containing HB-19 was applied on a 1-cm2 area of the CAM inside a silicon ring. After 48 hours of incubation at 37°C, CAMs were fixed in situ with saline-buffered formalin, excised from the eggs, placed on slides, and left to dry in air. Photographs were taken and the total length of the vessels was measured as described [47].

### Tumor cell inoculation in nude mice

All *in vivo* experiments were carried out with ethical committee approval and under the conditions established by the European Community. 4-week old female athymic nude Mice (Janvier, Le Genest St Isle, France) were injected subcutaneously in the right flank (MDA-MB-231) or in the mammary fat pad (MDA-MB-435) in 0.1 ml PBS. When the tumor reached about 40 mm^3^, the mice were separated randomly in several groups. Administration of HB-19 in PBS (5 mg/kg body weight) was either by intraperitoneal, subcutaneous or peritumoral injections three times per week. Tumor volume was measured along two major axes with calipers. Tumor volume (mm^3^) was calculated as follows: V = 4/3×π×R_1_^2^×R_2_, where R_1_ is radius 1, R_2_ is radius 2 and R_1_<R_2_.

### Tissue preparation, immunohistochemical staining and image analysis

Immediately after surgical resection, tumors, were frozen in liquid nitrogen and fixed for 20 min in acetone at 4°C or fixed with 4% PFA for paraffin inclusion. The sections of 8 µm were re-hydrated and saturated in PBS containing 1% BSA and 2% normal goat serum. To visualize tumor endothelial cells, sections were incubated with a Rat anti-mouse CD31 monoclonal antibody (1∶50) in saturation buffer 1 hour at room temperature. After two washes in PBS, sections were incubated for 1 hour at room temperature with biotinylated goat anti-Rat IgG (1∶1000 dilution; Chemicon International Inc., Temecula, CA) in saturation buffer, followed by three washes in PBS and incubation with the avidin-biotinylated-alkaline phosphatase complex (Vector Laboratories). Alkaline phosphatase activity was revealed using the Vector red substrate (Vector Laboratories). Finally, sections were counterstained with hematoxylin, followed by water wash and cover slipped with Thermo Shandon mounting medium (Pittsburg, USA). For each CD31-labeled section of control and treated tumors, five microscopic fields (200× magnification) containing exclusively viable tumor cells as indicated by hematoxylin staining were selected randomly for analysis. Angiogenesis was quantified by Image analysis of CD31-labeled endothelial cells. The EC density in each field was expressed as the ratio of EC area/total area examined ×100 (%). These values were then averaged for untreated (control) and treated tumors.

### Blood cell count

Blood samples were collected by cardiac puncture. Hematocrit level, white blood cells and platelets count were determined individually in whole blood anticoagulated with EDTA (1 mM) at room temperature. Blood cell count was analyzed with an automated flow cytometric blood cell counter (Cell-Dyn 3500, Abbott Laboratories, Rungis, France).

### Statistical analysis

The significance of variability between the results of each group and its corresponding control was determined by unpaired t-test and Mann-Witney Anova. All results are expressed as mean±standard errors of the means from at least two independent experiments.
